# Evaluating Literature Reviews Conducted by Humans Versus ChatGPT: Comparative Study

**DOI:** 10.2196/56537

**Published:** 2024-08-19

**Authors:** Mehrnaz Mostafapour, Jacqueline H Fortier, Karen Pacheco, Heather Murray, Gary Garber

**Affiliations:** 1 Canadian Medical Protective Association Ottawa, ON Canada; 2 Department of Emergency Medicine Queen's University Kingston, ON Canada; 3 Ottawa Hospital Research Institute Ottawa, ON Canada; 4 Department of Medicine and the School of Public Health and Epidemiology, University of Ottawa Ottawa, ON Canada

**Keywords:** OpenAIs, chatGPT, AI vs. human, literature search, Chat GPT performance evaluation, large language models, artificial intelligence, AI, algorithm, algorithms, predictive model, predictive models, literature review, literature reviews

## Abstract

**Background:**

With the rapid evolution of artificial intelligence (AI), particularly large language models (LLMs) such as ChatGPT-4 (OpenAI), there is an increasing interest in their potential to assist in scholarly tasks, including conducting literature reviews. However, the efficacy of AI-generated reviews compared with traditional human-led approaches remains underexplored.

**Objective:**

This study aims to compare the quality of literature reviews conducted by the ChatGPT-4 model with those conducted by human researchers, focusing on the relational dynamics between physicians and patients.

**Methods:**

We included 2 literature reviews in the study on the same topic, namely, exploring factors affecting relational dynamics between physicians and patients in medicolegal contexts. One review used GPT-4, last updated in September 2021, and the other was conducted by human researchers. The human review involved a comprehensive literature search using medical subject headings and keywords in Ovid MEDLINE, followed by a thematic analysis of the literature to synthesize information from selected articles. The AI-generated review used a new prompt engineering approach, using iterative and sequential prompts to generate results. Comparative analysis was based on qualitative measures such as accuracy, response time, consistency, breadth and depth of knowledge, contextual understanding, and transparency.

**Results:**

GPT-4 produced an extensive list of relational factors rapidly. The AI model demonstrated an impressive breadth of knowledge but exhibited limitations in in-depth and contextual understanding, occasionally producing irrelevant or incorrect information. In comparison, human researchers provided a more nuanced and contextually relevant review. The comparative analysis assessed the reviews based on criteria including accuracy, response time, consistency, breadth and depth of knowledge, contextual understanding, and transparency. While GPT-4 showed advantages in response time and breadth of knowledge, human-led reviews excelled in accuracy, depth of knowledge, and contextual understanding.

**Conclusions:**

The study suggests that GPT-4, with structured prompt engineering, can be a valuable tool for conducting preliminary literature reviews by providing a broad overview of topics quickly. However, its limitations necessitate careful expert evaluation and refinement, making it an assistant rather than a substitute for human expertise in comprehensive literature reviews. Moreover, this research highlights the potential and limitations of using AI tools like GPT-4 in academic research, particularly in the fields of health services and medical research. It underscores the necessity of combining AI’s rapid information retrieval capabilities with human expertise for more accurate and contextually rich scholarly outputs.

## Introduction

Artificial intelligence (AI) is a rapidly evolving technology that combines computer programming with large data sets to enable software to perform tasks. Generative AI uses this technology to synthesize content; the system is trained on large volumes of data to identify patterns until it can recognize those patterns and generate novel responses to queries. Large language models (LLMs), such as ChatGPT, are a form of generative AI wherein the software is trained on extensive textual data sets and can generate a response to prompts and questions [1].

AI in general and LLMs in particular are in a period of exponential growth, and researchers are exploring their utility to perform tasks with variable results [1-5]. Previous studies have shown how these tools can help to advance research [4]. One area where there is potential to realize efficiencies is in the creation of literature reviews and syntheses. The pace of scientific publication has been rapidly expanding [6], and AI tools may provide a useful starting point and substantial time savings by automating some elements of a literature search. However, there is little research that compares the results generated using AI with those generated by skilled human researchers.

The purpose of this study is to conduct a literature review using OpenAI’s ChatGPT-4 model (“GPT-4”) and then conduct a comparative analysis against a review conducted by human researchers.

The way researchers use these tools and optimize the language used to generate a response from ChatGPT, known as prompt engineering, directly impacts the quality of results [7]. Clear, concise, neutral, structured, and specific prompts reduce the model’s tendency to respond with generic or off-topic responses, as well as generate an unsubstantiated or false response, also termed an AI hallucination [8]. Therefore, in order to conduct this study, we have introduced an approach to prompt engineering that may assist researchers who wish to use GPT-4 or other LLMs to generate literature reviews.

## Methods

### Overview

We started with a completed literature review exploring the factors influencing the relational dynamics between the physician and the patient that motivate patients to file medicolegal complaints against physicians [9]. Using this review as a reference standard, we then tasked Open AI’s GPT-4 model (training data updated in September 2021) with producing a literature review on the same topic. Subsequently, we compared the results generated by GPT-4 and the literature review conducted by human experts. It should be noted that while GPT-4 was used to generate a literature review and make suggestions for the paper title, it was not used to write this paper.

### Human Literature Review

The first author conducted a traditional literature review to identify what factors affect relationships between physicians and patients. They used a systematic approach to ensure transparency and reproducibility. The review included a mix of studies and assessed both qualitative and quantitative data together through thematic analysis [10]. With the help of a research librarian, they developed a search strategy using Medical Subject Headings (MeSH) terms, keywords, and key phrases for a single database (Ovid MEDLINE) to identify articles related to physician-patient relationships. The search strategy was calibrated to identify articles that were most relevant to the research question, rather than prioritizing an approach that would capture every potentially relevant paper (detailed in the “Search strategy developed for literature search led by human researchers” section in Multimedia Appendix 1).

**Figure 1:**
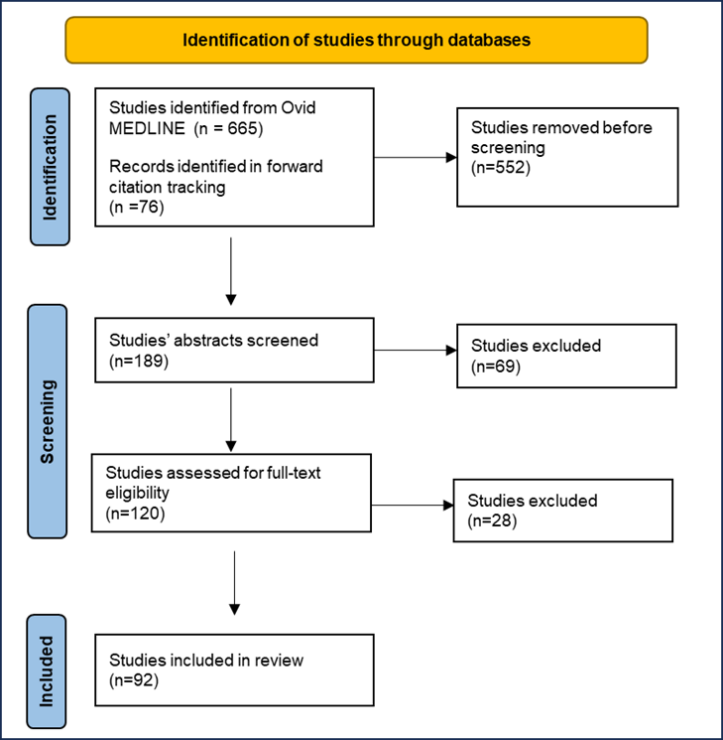
Overview of article screening and inclusion into the study.

Subsequently, the librarian screened titles and abstracts, and then the main author screened full-text papers for inclusion against predefined eligibility criteria. Papers had to be empirical research studies or literature reviews that discussed relational factors between physicians and patients that affected patient satisfaction and medicolegal complaints. Studies were excluded if they were not based on empirical research (eg, editorials, commentaries, and reports) or if they were unrelated to the research question (Textbox 1; Figure 1).

**The eligibility criteria to identify relevant studies.**

**Inclusion criteria**

- The study described an empirical research study or a literature review.
- The study focused on or described relational factors between physicians and patients impacting patients’ satisfaction.
- The record focused on or described the relationship between patients’ satisfaction and medicolegal risk against physicians.
- The study focused on or described medicolegal complaints against physicians caused by relational problems between patients and physicians.

**Exclusion criteria**

- The study was not empirical research, for example, editorials, commentaries, and reports.
- The study contained no explicit mention of physicians and patients’ relationships.
- The study was not related to either patients’ satisfaction or medicolegal risk against physicians.

We used a thematic analysis approach to review and synthesize the included manuscripts to identify the relational factors that influence patient satisfaction and medicolegal complaints, and we reported the findings in a published narrative review [9].

### AI Prompt Engineering for the Literature Search

Previous work suggested that the use of single prompts may not be very effective for complex tasks [2]. We began our process with a single prompt for the literature search (detailed in the “Single Prompt” section in Multimedia Appendix 1), and the results were clearly inadequate, confirming these findings. Consequently, we developed a series of prompts in an iterative, sequential format. This approach operated on the premise that GPT-4 would benefit from incremental and iterative guidance to yield optimal results. In this approach, the researcher designed sequential prompts based on the assessment of the previous responses generated by GPT-4, starting from a general prompt, and designing subsequent prompts to refine the output toward the desired form.

An initial series of prompts was used to explore GPT-4’s breadth of knowledge about the factors impacting relationships between physicians and patients. The first prompt was general, simple, and short, asking GPT-4 to list relevant factors to the subject matter. Since we did not know all the relevant factors related to the topic, subsequent prompts were designed to ask for more factors to extend the list of factors and reinforce previous instructions while specifying the desired tone. Further prompts extended the factors and ensured the validity of their content by introducing additional criteria such as the number of sentences and asking for precise references (Table 1; detailed in the “Identifying relational factors” section in Multimedia Appendix 1).

**Table 1:** Iterative prompts used to generate contributing factors.

Step	Prompts
1	Write a literature review on the relational problems between physicians and patients that lead to medicolegal complaints against physicians, from a health service researcher perspective, and provide precise references.
2	Please add at least 15 more factors related to relational problems between physicians and patients that lead to medicolegal complaints against physicians to the list, considering the sensitivity, precision, and accuracy of information.
3	You provided me with 21 relational factors between physicians and patients that contribute to the likelihood of filing a medicolegal case against a physician. Please write an elaborated, scientific, and accurate description for each factor that includes at least 15 sentences, and provide at least two real and precise references that support your arguments.

In order to replicate the format of the literature review done by human researchers, the researcher who had conducted the literature review explored a series of prompts to guide GPT-4 through a more in-depth exploration of the identified relational factors. They began by prompting GPT to suggest evidence-based ways to improve each relational factor (eg, “please also describe how to address communication issues using methods derived from scientific publications and research”), which were then evaluated. In cases where the proposed strategy was deemed unsuitable, they either recommended a specific alternative to replace the initial strategy or asked ChatGPT-4 to generate a different one. If the new strategy met the criteria, they instructed GPT-4 to incorporate it into the written description. For example, when asked about communication issues, GPT-4 first suggested the Four Habit Model [11], which they evaluated to be somewhat out of date. With further prompting, GPT-4 suggested newer strategies to improve communication between physicians and patients, such as the teach-back method and the Shared Decision-Making Model [12], which they then instructed GPT-4 to incorporate in the description. They were able to make these adjustments because they used ChatGPT search while armed with subject matter expertise and an understanding of the available literature for this topic. They leveraged this knowledge to refine the approach to prompt engineering during the process (Table 2; detailed in “Exploring communication as a factor” in Multimedia Appendix 1).

**Table 2:** Iterative prompts used to elaborate on each factor.

Step	Prompts
1	Please also describe how to address communication issues using methods derived from scientific publications and research.
2	Is the Four Habit Model the most cited and most recent paper on how to address communication problems? Can you please find a balance between the most cited research papers and the most recent ones, when trying to find references to explain the problem and to address the problem?
3	Please explain the teach-back model and shared decision-making in communication issues using relevant references.

### Comparison of Human Versus AI Literature Reviews

To the best of our knowledge, there are no validated tools or checklists to compare human and AI literature reviews. Therefore, we chose to compare the reviews subjectively with respect to the accuracy, response time, comprehensibility, consistency, breadth and depth of knowledge, contextual understanding, and transparency of the outputs. The criteria are defined as follows:

1. Accuracy: we defined the accuracy of the outcome as the percentage of correct responses.
2. Response time: we defined response time as the time it took to conduct the review, including identifying factors and demonstrating what they are.
3. Consistency: we defined consistency as the degree of reliability and stability in the results of a study when it is repeated under similar conditions to ensure it can be replicated.
4. Breadth of knowledge: we defined the breadth of knowledge as the extent and range of information one has access to across various subjects and disciplines.
5. Contextual understanding: contextual understanding refers to the ability to comprehend the meaning and relevance of information within its specific context. It goes beyond just the literal meaning of words but also the nuances and implications shaped by the situation and the specific circumstances under which the communication occurs [13].

### Ethical Considerations

All the studies done at the Canadian Medical Protective Associations have received ethics approval from the ethics review panel of the Advarra Institutional Review Board (Protocol #00020829).

## Results

### Exploring Literature Search Using AI (GPT-4 Model)

Using iterative prompts and starting from a general prompt, GPT-4 initially generated 6 relational factors. After being repeatedly asked for more factors, it became evident that GPT-4 began to produce unrelated factors after reaching 21, likely due to hallucinations. Overall, GPT-4 generated a list of 21 relational factors and provided 54 references (Table 3). Of these 21 factors, 14% (n=3) were identified as irrelevant. About 24% (n=13) of the references were identified as somewhat related to the topic but not particularly strong in their relevance, and 7.5% (n=4) were identified as irrelevant.

**Table 3:** Factors affecting the relationships between physicians and patients that lead to patient dissatisfaction and medicolegal complaints as identified in human- and artificial intelligence (AI)–led literature reviews.

Human literature review	AI-generated literature review, following iterative prompts 1-3
Communication Understanding patients’ concerns and expectations Clarity of communication Information sharing and transparency Tone and attitude Individual characteristics Physician characteristics Patient characteristics Perceived care and empathy Health care system and policies Navigating the health care system Wait times Resource constraints	1. Communication issues 2. Lack of informed consent 3. Perceived negligence or incompetence 4. Mismatched expectations 5. Perceived lack of care or empathy 6. Systemic issues 7. Cultural and language barriers 8. Failure to follow-up 9. Breakdown in continuity of care 10. Patient autonomy disregarded 11. Trust erosion 12. Financial conflicts of interest 13. Power dynamics 14. Failure to respect confidentiality 15. Inadequate documentation^a^ 16. Unaddressed patient concerns 17. Provider burnout 18. Poor coordination among care teams^a^ 19. Patient’s previous negative experiences 20. High patient expectations 21. Medical complexity^a^

^a^Factors indicated with an asterisk were identified by GPT-4 but were judged to be inaccurate by human researchers.

GPT-4 demonstrated an impressive ability to retrieve a breadth of information; however, our assessment showed that this information could be superficial, requiring an in-depth investigation to ensure its reliability and validity. Since we were uncertain how many relevant factors were related to the topic, we prompted GPT-4 to extend the list of relevant factors. We also observed that GPT-4 will not communicate to its users when the topic has been saturated or when to stop asking for more information. For example, when we pushed it to go beyond contributing to the relational problems between physicians and patients, GPT-4 provided 30 factors, but the additional factors were increasingly irrelevant or obviously incorrect.

Although the description provided by GPT-4 for each factor was initially short and concise, with prompting, the elaborations for each factor became more detailed and comprehensive. In addition, we noted that GPT-4 initially displayed limitations in adhering to prompted numerical guidelines, such as requesting a specific sentence count, word limit, or number of references, but it started to better follow the instructions when they were reinforced in subsequent prompts.

Our findings showed that GPT-4 can offer relevant responses to questions; however, there were instances where more precise, suitable, or applicable alternative answers existed. For example, when tasked with suggesting a mitigation strategy for communication issues between physicians and patients, GPT-4’s initial recommendation was the Four Habits Model. However, upon deeper expert analysis, the researchers determined that the teach-back method and Shared Decision-Making Model were more fitting for the review. This underscores that initial responses from GPT-4, although relevant, may require further evaluation to determine their optimal relevance and applicability.

### Human Literature Review

A total of 120 articles were identified for review. Title and abstract screening against the eligibility criteria yielded 113 papers that were directly relevant to our objectives, of which 92 were included for full-text reading and analysis. Two researchers (MM and JHF) reviewed the included articles and identified factors that affected the physician-patient relationship in ways that contributed to patient satisfaction, dissatisfaction, and potential medicolegal complaints. These factors were sorted into the themes and subthemes displayed in Table 3.

### Comparison of Human Versus AI Literature Reviews

#### Overview

**Figure 2:**
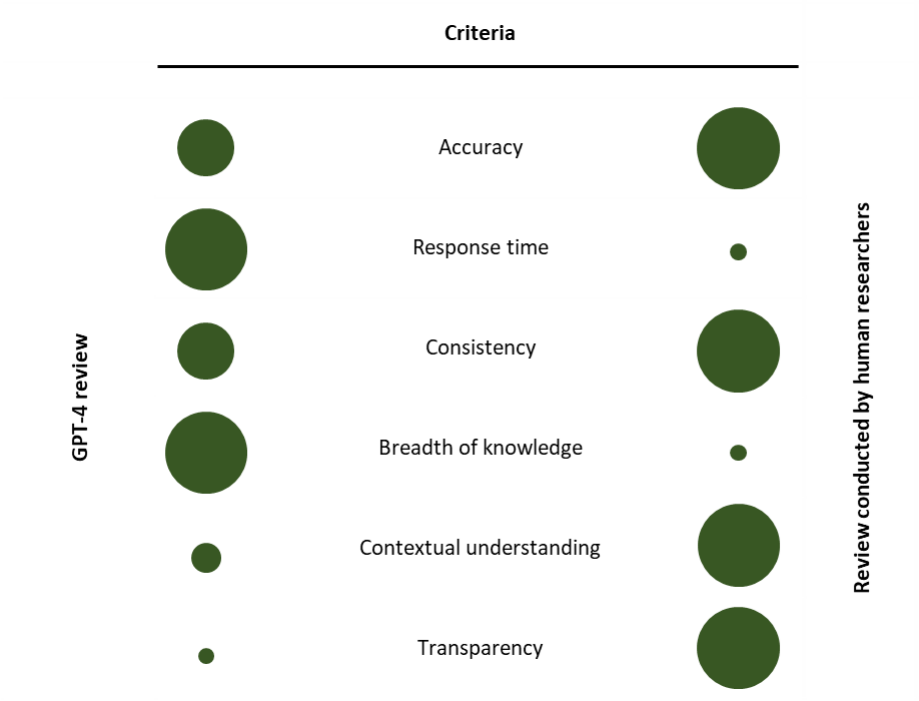
Qualitative comparison of reviews conducted by GPT-4 versus. human researchers. Circle sizes (large to small) qualitatively represent differences in criteria between GPT-4 and human researchers; they are not intended for precise measurement.

While both reviews identified factors influencing the relational problems between physicians and patients, there were important differences. In the human-led literature search, we used a semistructured approach to find relevant references, then conducted a thematic analysis to group the factors into themes and convey the concepts clearly to the target audience. In contrast, GPT-4 used a proprietary search algorithm to explore the web, find relevant articles, and identify relevant factors. Also, it only followed the instructions to list the factors, so there was no synthesis or grouping of the factors. A qualitative comparison of the categories below can be found in Figure 2.

#### Accuracy

Of the 21 relational factors that were produced by GPT-4, 86% (n=18) were assessed to be accurate based on our subjective assessment (Table 3; detailed in the “Identifying relational factors” section in Multimedia Appendix 1). As noted above, GPT-4 will continue to suggest factors based on the user’s prompting, so the accuracy rate would decline if the user kept asking for additional factors.

In contrast, experienced human researchers have the nuanced judgment to identify the relevant factors and eliminate the ones that are not relevant to the subject matter. Typically, they can offer a coherent rationale to justify their identification of a factor as either relevant or irrelevant to the subject matter. In the review conducted by researchers, all the identified factors are considered quite relevant to the subject matter, and their relevance is supported by scientific evidence.

#### Response Time

The AI model generated results within seconds, and the entire series of experiments and prompts were conducted over a few days. The human-led literature review was not conducted as a time trial and occurred as part of a researcher’s regular activities over the course of several months. Had the review been conducted explicitly for this study, it would have required substantially more time for human researchers to search the literature, read and comprehend the papers, and produce results, compared with GPT-4. Our evaluation indicated that OpenAI’s GPT-4 model demonstrated an unparalleled advantage in response time.

#### Consistency

In general, the GPT-4 model produced reliable responses to prompts, but similar prompts could sometimes result in variable outputs. We observed that shorter and more precise prompts were more likely to yield consistent results, whereas complexity and length in prompts led to more variability in outputs. When conducting literature reviews, human researchers produce fairly consistent results when they have adequate resources (eg, access to a skilled health research librarian for literature search strategies) and follow established techniques (eg, PRISMA [Preferred Reporting Items for Systematic Review and Meta-Analysis] for systematic reviews) [10].

#### Breadth and Depth of Knowledge

Our experiments demonstrated a considerable breadth of knowledge within the GPT-4 model, significantly surpassing that of human researchers. This was particularly evident when the model almost instantly generated an extensive list of contributing factors to relational problems between physicians and patients, as well as a comprehensive list of potential mitigation strategies for each factor.

While breadth of knowledge is valuable when conducting literature reviews, synthesizing the information derived from such a review requires deep knowledge and the ability to apply, analyze, and evaluate information related to that topic. This is an area where the GPT-4 model fell short, and a human researcher with experience in a specific subject area may have an advantage.

#### Contextual Understanding

While LLMs are nonsentient and do not understand meaning in a traditional sense, our experiments revealed that GPT-4 was able to produce outputs that included a satisfactory level of contextual information to allow readers to understand and link key concepts. For example, through iterative prompting, the software was able to produce a list of physician-patient relational issues that included factors as varied as power dynamics, provider burnout, medical complexity, and cultural and language barriers. This level of context was improved by iterative feedback and prompting, providing expanded definitions and additional references [13]. However, given the fact that GPT-4 started to hallucinate when asked to generate more factors, we concluded that it did not have a deep contextual understanding to stop generating meaningless outcomes. On the other hand, human researchers possess an understanding of meaning that consistently results in superior proficiency in interpreting and responding to nuanced contextual elements in this literature search, which would prevent such errors.

#### Transparency

Another area where human researchers have an advantage is transparency. Human researchers can describe their literature search methods, state and rationalize eligibility criteria, explain the inclusion or exclusion of various articles, describe the approaches used in synthesis, and answer specific questions about their methods. There is significantly less transparency in the way that LLMs process prompts, collect information, and generate outputs at this time. Even when prompted to explain how it completed its literature review, GPT-4 will explain broadly that it drew upon diverse training data but cannot provide a full list of the relevant resources it reviewed, and so the backend review process is almost hidden.

## Discussion

### Overview

Many researchers are considering how AI tools can support their research. As with any new technology, there is a spectrum of uptake from “early adopters” to “stubborn resistors.” This paper explored how a widely available LLM tool, GPT-4, conducts literature reviews and compares the generated outcomes with a similar review conducted by human researchers.

We found that human-generated literature reviews were more transparent, consistent, and accurate, as long as the literature review was approached systematically and the researcher had sufficient experience and expertise in the subject area. In contrast, GPT-4–generated results were much faster, provided an impressive breadth of content, and were reasonably accurate. We also found that the model was often inconsistent in its outputs and at times generated irrelevant information, especially if forced to generate a certain number of factors.

One of the fundamental differences between the literature review generated by GPT-4 and humans was in terms of contextual understanding. We attribute this difference to one often-cited limitation of LLMs: their status as so-called “stochastic parrots” [14] that use statistical probabilities of which word is most likely to be next rather than understanding meaning. With prompting, GPT-4 rapidly produced an extensive list of factors that affect the relationship between physicians and patients that appeared very relevant. However, a deeper examination by experts identified inaccurate outputs among accurate ones. This underscores the necessity of expert evaluation in discerning the nuanced veracity of the information generated by GPT-4.

In fact, in this study, we identified 2 potential scenarios where researchers might encounter challenges while working with GPT-4. First, effective communication with the model, specifically through adept prompt engineering, is crucial. Inadequate or improper prompting, particularly for complex tasks like conducting a literature review, leads to unsatisfactory results (detailed in the “Single prompt” section in Multimedia Appendix 1). Second, novice researchers, unfamiliar with a specific field, might use effective prompting techniques and obtain a broad array of information. This breadth of knowledge can be initially impressive, yet it is important to recognize that the generated content may include errors or inaccurate information. It is for this reason that researchers must carefully review the results to identify and correct potential inaccuracies. The importance of expert oversight in evaluating the reliability of GPT-4–generated content is clear.

This paper introduces an iterative algorithm to effectively search the literature to address the first challenge. We suggested an approach to prompt engineering that uses directive iterative prompts to guide GPT-4 to develop a literature review for researchers. This structured approach includes 2 phases. In the initial phase, researchers are advised to formulate a sequence of prompts that is broad yet precise, progressively becoming more specific. This approach should be designed to incrementally introduce and reinforce instructions, guiding GPT-4 toward generating an output that offers a thorough and comprehensive perspective on a particular subject. In the second phase, the researcher can independently query elements, concepts, or factors identified in the first phase to explore these in greater detail. At all phases of the process, the researcher’s own understanding of the subject will shape the prompts and drastically improve GPT-4’s literature review, suggesting relevant ideas and references while guiding the software away from outdated or incorrect concepts.

We suggest approaching GPT-4 as a research assistant who possesses limited contextual expertise and occasionally synthesizes responses entirely to overcome the second challenge. This requires substantial insight and knowledge from the researcher to diligently guard against the so-called “hallucinations” of the software. Such vigilance is crucial, as GPT-4 can produce convincing yet entirely fabricated content and references [2,15].

For this reason, it seems that GPT-4 might be a more useful tool for experienced researchers looking for wide surveys on a particular topic. The human researcher’s knowledge and expertise in a specific area allows them to develop appropriate prompts, iterate with the software to refine the outputs, introduce relevant frameworks and key references, and ultimately guide the process toward the desired output with a clear-eyed understanding of the limitations of what is produced. However, it can also offer different benefits to other audiences, including more novice researchers. Leveraging its extensive knowledge base and inhuman quickness, GPT-4 can help newcomers familiarize themselves with the domain under review. The software acts as an information assistant, offering a wide spectrum of knowledge within a defined domain. In addition, for researchers who have few resources or constrained schedules, it can be used to facilitate the literature review process by offering a robust preliminary draft outline, encompassing key concepts that serve as foundational building blocks. Other studies have explored the potential use of GPT-4 and other LLMs for research tasks such as scholarly writing [2,16], medical writing [15,17,18], and systematic reviews [19]. Still, the rapid improvement in generative AI software has also spurred rapid growth in concerns, such as those related to the ethics of ChatGPT as a coauthor [20] or the potential for it to be used to disseminate misinformation and promote plagiarism [4]. As with any nascent technology, transparency around its use will be essential, and caution is perhaps warranted.

Overall, this study clearly demonstrates the potential utility of GPT-4, an LLM, in supporting the conduct of literature reviews, particularly when an iterative feedback approach to prompt engineering is used. The software successfully reviewed the literature, identified several factors relevant to the subject matter, and was able to respond to prompts requesting additional detail and references. In some instances, and for some researchers, the benefits of using GPT-4 for a literature review (including good breadth of knowledge, reasonable accuracy, and an impressive response time) outweigh the identified shortcomings (including some inconsistency, some inaccuracy, and less depth of knowledge). We suggest that our structured approach to prompt engineering may serve as a model for researchers looking to integrate generative AI into their literature searches. Given the detailed assessment of the generated outcomes with human-led reviews, we recommend approaching these models as an assistant rather than a wise professor; researchers relying on GPT-4 to provide them with a full and nuanced understanding of a complex or rapidly-evolving subject do so at their own peril.

### Limitations and Future Research

This study has some limitations. Given the iterative nature of our approach to prompting GPT-4, we did not predefine our prompts or methods, and the researcher leading the prompts (MM) had extensive experience in the subject area; these factors undoubtedly influenced our prompts and thus our outcomes. Our approach to comparing the human- and AI-led literature reviews was subjective, exploratory, and qualitative.

We acknowledge the limitations posed by using a single database and using a human-conducted review as the comparison standard. However, the opaque nature of ChatGPT’s search strategy presents challenges in directly comparing search methodologies. These aspects are critical for interpreting our findings and suggest avenues for future research. In addition, while we have detailed GPT-4’s prompt strategies in the Multimedia Appendix 1, the proprietary and evolving nature of its algorithm limits a comprehensive methodological comparison. Future research should examine AI capabilities in detecting emerging trends and gaps, enhancing our understanding of its utility and constraints in academic research.

In our methodology for the human literature search, we used thematic analysis, a subjective process influenced by the researchers’ expertise and perspectives. We highlight the inherent subjectivity of thematic analysis as a key limitation. Similarly, our review of ChatGPT’s capability to conduct literature reviews acknowledges the qualitative and subjective nature of this evaluation. Our aim was to offer insights and guidance for researchers interested in leveraging AI tools like ChatGPT in their research endeavors.

This study’s methodology involved the same researcher in both conducting the human literature review and guiding the AI, as well as participating in the team that evaluated the outcomes. While this was intended to leverage the researcher’s subject expertise, it introduces a potential bias, as the researcher was not blinded to the results of the human review during the AI evaluation. This could influence the assessment and interpretation of the AI-generated content. Future studies might consider a more diversified evaluation team to further mitigate bias and enhance the objectivity of the findings.

This study is limited to an in-depth examination of the ChatGPT-4 model, providing a detailed understanding of this specific tool’s capabilities and limitations in conducting literature reviews on a particular topic. While this focus allows for a precise evaluation of GPT-4, we acknowledge that this technology is evolving very fast, and it may not reflect the performance of other AI tools that are designed to handle similar tasks. Despite this limitation, our work shows the potential of AI to streamline the initial stages of literature reviews. To build on this foundation, future research should compare the effectiveness of various AI models across a broader range of topics, thereby enhancing our understanding of the general applicability of AI-assisted literature reviews.

Moreover, upcoming studies should focus on enhancing prompt engineering methods to further leverage ChatGPT-4’s capabilities in conducting literature reviews. Addressing identified limitations, such as improving the depth and contextual understanding of AI-generated reviews, is crucial. Expanding the training data sets of ChatGPT-4 to include more diverse and recent publications could potentially mitigate issues of relevance and accuracy. In addition, investigating the role of AI in identifying emerging trends and gaps within specific research fields, particularly in health services and medical research, would provide valuable insights into the practical applications and limitations of AI in academic research.

### Final Notes and Considerations

In incorporating AI such as ChatGPT into academic research, ethical considerations are crucial. There is the potential for bias in AI outputs, reflecting the biases present in the training data. Ensuring transparency about how AI is used, including prompt selection and response interpretation, is vital for replicability and trust. Responsible use of AI requires acknowledging its limitations and not substituting it for human expertise. As AI technologies become more prevalent in research, it is essential to establish ethical guidelines that promote awareness of bias, transparency, and responsible usage. Integrating ChatGPT-4 and similar LLMs into academic research could dramatically change how we conduct studies, particularly literature reviews. This technology could speed up our ability to study extensive fields, enabling quicker responses to new information or gaps in knowledge. However, it is crucial to remember that the depth of understanding and critical analysis, which are at the heart of academic work, cannot be fully replicated by AI.

The use of LLMs might also make research more accessible, allowing a wider range of voices to contribute to scholarly conversations. Yet, we must navigate this future carefully, paying close attention to ethical concerns like bias in AI outputs and maintaining transparency in AI’s role in research processes. As we move forward, the challenge will be to harness AI’s power to enhance our work while ensuring that the essence of research, critical thinking, depth of analysis, and human insight remain at the forefront. The potential is vast, but it is also our responsibility to use these tools wisely.

## Appendix

Multimedia Appendix 1 (A) Single prompt. (B) Identifying relational factors. (C) Exploring communication as a factor. (D) Search strategy developed for literature search led by human researchers.

## Abbreviations

AI: artificial intelligence
LLM: large language model
MeSH: Medical Subject Headings
PRISMA: Preferred Reporting Items for Systematic Review and Meta-Analysis
